# Risk factors and mediating mechanisms of restless legs syndrome in patients undergoing maintenance hemodialysis: a longitudinal cohort study combined with Mendelian randomization analysis

**DOI:** 10.3389/fneur.2026.1819747

**Published:** 2026-07-02

**Authors:** Shuge Yao, Yucai Zhang, Chenghong Ma, Huixin Wen, Zhenxia Huo, Yucong Zhou, Liang Wu

**Affiliations:** 1 Department of Radiology, The Second Affiliated Hospital of Xingtai Medical College, Xingtai, China; 2 Department of Nephrology, The Second Affiliated Hospital of Xingtai Medical College, Xingtai, China

**Keywords:** latent growth curve mediation model, longitudinal cohort study, maintenance hemodialysis, Mendelian randomization for mediation analysis, multivariable generalized estimating equations, restless legs syndrome

## Abstract

**Background:**

Somatic wasting and psychological distress frequently cluster in patients on maintenance hemodialysis (MHD), but their longitudinal joint trajectories and germline-level causal pathways with downstream dopamine metabolism in restless legs syndrome (RLS) remain unclear.

**Methods:**

This multi-stage study combined a prospective cohort and genetic triangulation. First, within a matched 5-year longitudinal MHD cohort (*N* = 192), multivariable generalized estimating equations (GEE) and latent growth curve mediation models (LGCM) quantified time-dynamic risks and decomposed trajectory mediation pathways. Findings were validated via landmark diagnostics and replicated in the full unmatched imputed cohort (*N* = 321). Second, bidirectional and network two-sample Mendelian randomization (MR) utilized independent GWAS statistics to map genetically predicted renal impairment (eGFR/UACR) against plasma dopamine 3-O-sulfate levels via psychological and musculoskeletal traits.

**Results:**

Longitudinal GEE revealed that sarcopenia (Adjusted OR = 1.58, *p* = 0.002) and depressive severity (Adjusted OR = 1.10, *p* < 0.001) independently predicted 5-year incident RLS, compounded by a significant multiplicative interaction (OR = 1.50, *p* < 0.012). Decoupled LGCMs showed that chronic trajectories of depression, anxiety, frailty, and sarcopenia mediated 44.4, 45.5, 45.8, and 46.2% of the total risk pathways, respectively (all *p* < 0.001). MR triangulation confirmed that lower eGFR causally associated with increased depression risk (IVW: IVW: *β* = −0.579, *p* = 0.004), which directionally mediated a substantial indirect effect on suppressed dopamine 3-O-sulfate metabolism (indirect effect: 0.401, *p* = 0.004; accounting for 41% of the total effect), whereas anxiety lacked mediating reliability.

**Conclusion:**

This study establishes a stable somatopsychic-neurological cascade where chronic phenotypic deterioration and genetically predicted depression mediate the pathway from renal impairment to suppressed dopamine metabolism, highlighting multi-system targets for early RLS screening and preventive intervention.

## 1 Introduction

Restless legs syndrome (RLS) is a prevalent sensorimotor disorder behaviorally characterized by an irresistible urge to move the lower extremities, typically accompanied by uncomfortable or distressing paresthesia that unremittingly worsens during periods of rest or nocturnal hours. These distressing manifestations profoundly disrupt sleep architecture, compromise daytime cognitive and physical functioning, and ultimately degrade overall health-related quality of life (1, 2). While the global prevalence of RLS in the general adult population is estimated at approximately 3% (3, 4), this burden escalates dramatically in patients with chronic kidney disease (CKD), particularly those undergoing maintenance hemodialysis (MHD), where systematic reviews report a disproportionate prevalence shifting between 15 and 30% (4, 5). Advanced renal impairment precipitates a complex milieu of uremic toxin accumulation, persistent microinflammation, and disrupted bone-mineral pathways, all of which are intricately intertwined with the pathogenesis of RLS. Methodologically, iron deficiency and altered systemic iron homeostasis common in MHD impair iron transport across the blood–brain barrier, destabilizing tyrosine hydroxylase activity and compromising both central and peripheral dopaminergic signaling cascades (6–8).

Compounding this neurobiological vulnerability, MHD patients routinely experience profound, overlapping clinical deterioration spanning both somatic and affective domains, manifesting as sarcopenia, frailty, depression, and anxiety. Somatic wasting phenotypes (sarcopenia and frailty) and somatopsychic distress (depression and anxiety) frequently cluster, driven by shared pathways of chronic systemic wasting, neuroendocrine dysregulation, and functional malnutrition (9–12). Despite their clinical ubiquity, the vast majority of existing RLS research in the dialysis population relies heavily on cross-sectional frameworks. These static designs are inherently inadequate for establishing temporal sequencing, capturing time-dynamic trajectory variations, or isolating the cumulative longitudinal burden of these evolving multi-system phenotypes on incident RLS development. Furthermore, the downstream causal pathways connecting progressive renal insufficiency with disrupted dopamine metabolism via these somatopsychic intermediate phenotypes remain critically unmapped. Observational frameworks are perpetually vulnerable to residual unmeasured confounding and reverse causation, hindering a granular understanding of the structural mediation networks (13, 14).

To resolve these critical methodologic and mechanistic gaps, this study constructed a multi-stage prospective framework leveraging an integrated dual-channel design. First, using a 5-year longitudinal MHD cohort, an optimal multivariate clinical matching algorithm and longitudinal generalized estimating equations (GEE) were executed to control for fundamental baseline demographics and dialysis infrastructure parameters, thereby quantifying the independent and synergistic time-dynamic interaction effects of the four clinical phenotypes on 5-year incident RLS. Landmark analyses were applied to verify early prediction windows, and decoupled latent growth curve mediation models (LGCM) were formulated to decompose the longitudinal mediation potencies of phenotypic intercepts and slopes. Second, to triangulate these observational findings at the germline level and minimize environmental confounding, we implemented a bidirectional and network mediation two-sample Mendelian randomization (MR) analysis using independent genome-wide association study (GWAS) statistics. By mapping genetically predicted estimated glomerular filtration rate (eGFR) and urinary albumin-to-creatinine ratio (UACR) against plasma dopamine 3-O-sulfate levels, we systematically evaluated whether and to what extent renal impairment causally drives dopaminergic metabolic suppression through the directional mediation of psychological and musculoskeletal traits. Collectively, this study aims to provide a holistic, time-dynamic, and genetically validated multi-system paradigm of RLS development, offering robust theoretical targets for subsequent screening and preventive therapeutic strategies.

## 2 Materials and methods

### 2.1 Overall study design

**Figure 1:**
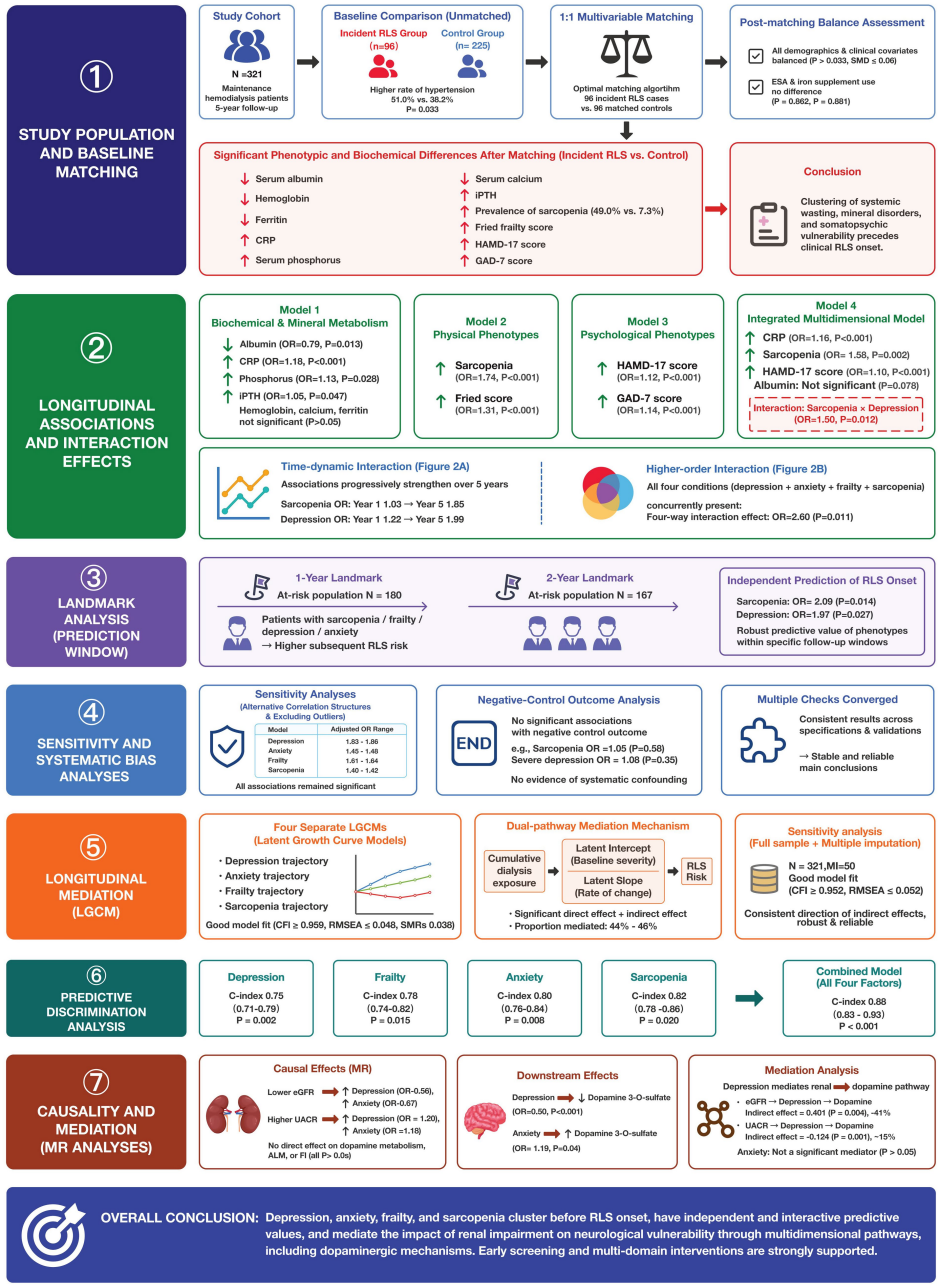
Graphical overview of the study design, longitudinal analyses, mediation modeling, and predictive framework for incident RLS in MHD patients. This schematic summary illustrates the overall analytical framework and major findings of the present longitudinal study investigating multidimensional phenotypic predictors and mechanistic pathways associated with incident RL in MHD patients.

This study employed a multi-stage, longitudinal observational framework coupled with a two-sample bidirectional and network mediation MR design (Figure 1). The central objective was to elucidate the time-dynamic prospective associations, higher-order multi-way phenotypic interactions, and underlying genetically predicted causal pathways linking renal function parameters, somatopsychic phenotypes (depression, anxiety, frailty, and sarcopenia), and downstream dopaminergic metabolism with the 5-year incidence of RLS in patients undergoing MHD.

The investigation was executed in three sequential analytical phases:

Phase I: Clinical Baseline Homogenization and Observational Cohort Establishment. To rigorously isolate the predictive value of target clinical phenotypes and biomandatory markers, a 1:1 clinical exact matching protocol was applied to the study population at baseline. Patients who subsequently transitioned to incident RLS during the 5-year longitudinal follow-up were strictly matched with controls based on identical demographic backgrounds and dialysis infrastructure trajectories to eliminate fundamental confounding.
Phase II: Longitudinal Prediction, Interaction Mapping, and Structural Mediation. Utilizing the homogenized prospective cohort, GEE were constructed to estimate the longitudinal risk trajectories and time-dynamic multiplicative interactions (ranging from two-way synergy to four-way pan-phenotypic clustering) over the 5-year follow-up. Landmark analyses at fixed 1-year and 2-year milestones were implemented to verify the early-screening stability of the individual risk axes. Subsequently, decoupled LGCM models were constructed under strict sample-to-parameter ratio constraints to decompose the dual-channel longitudinal mediation potencies (latent intercepts vs. latent slopes) of phenotypic progression.
Phase III: Genetic Triangulation and Network Causal Cascade Inference. To validate the observational findings at the germline level and minimize residual confounding or reverse causation, a bidirectional two-sample MR was conducted using independent GWAS summary statistics. Finally, a network MR mediation analysis was implemented to reconstruct the causal cascade, evaluating whether and to what extent the genetic liability of renal impairment (eGFR and UACR) impacts downstream dopaminergic metabolism (dopamine 3-O-sulfate) via the directional mediation of psychological health indicators.

The observational component of this study was conducted in strict accordance with the Declaration of Helsinki. All participants provided written informed consent prior to enrollment, and the study protocol was approved by the institutional review board.

### 2.2 Study population and selection criteria

This study systematically recruited adult patients undergoing MHD at a single clinical center between August 2020 and August 2025.

To be eligible for inclusion, participants were required to meet the following criteria:

- Aged ≥ 18 years.
- Receiving regular hemodialysis for a minimum duration of three consecutive months with demonstrated hemodynamic stability.
- Presenting with an absolute absence of RLS symptoms at baseline, alongside the physical and cognitive capacity to complete all required laboratory tests, physical performance metrics, and somatopsychic questionnaires.
- Providing signed written informed consent prior to enrollment.

Patients were excluded if they met any of the following pre-specified criteria:

- Severe active cardiovascular or cerebrovascular diseases, including New York Heart Association class III–IV heart failure, acute myocardial infarction, or recent stroke;
- Pre-existing primary neurological disorders or significant peripheral neuropathies that could confound RLS diagnostics, such as Parkinson’s disease or severe advanced diabetic neuropathy;
- Major psychiatric or cognitive impairments impeding reliable self-reported symptom scales;
- Current use or recent exposure to medications known to modulate RLS symptoms, including dopamine receptor agonists, or long-term therapeutic dependence on sedative–hypnotic agents that could potentially mask subclinical RLS manifestations.

The investigation was conducted in strict compliance with the tenets of the Declaration of Helsinki. The study protocol was reviewed and formally approved by the institutional ethics committee (Approval No. KY2021028).

### 2.3 Study design, variable specification, and follow-up

This investigation employed a single-center, prospective longitudinal cohort design with a 5-year follow-up period spanning from August 2020 to August 2025. The primary prospective endpoint was defined as the incident onset of RLS, systematically adjudicated annually according to standard clinical diagnostic criteria.

A comprehensive, multi-dimensional battery of clinical parameters was rigorously collected at baseline (*T_0_*) and updated through structured annual follow-up waves (*T_0_* to *T_1_*):

Demographics and Dialysis Infrastructure Backgrounds: Age, sex, body mass index (BMI), presence of key comorbidities (e.g., hypertension, diabetes), dialysis vintage, vascular access type (arteriovenous fistula [AVF] utilization), dialysis adequacy (quantified via urea reduction kinetics, Kt/V), and cumulative systemic comorbidity burden evaluated using the Charlson Comorbidity Index (CCI). Baseline therapeutic exposures including erythropoiesis-stimulating agents (ESA) and iron supplement utilization were also recorded.
Laboratory Biomarkers and Mineral Metabolism Biomarkers: Serum albumin, hemoglobin, ferritin, C-reactive protein (CRP), serum calcium, serum phosphorus, and intact parathyroid hormone (iPTH) levels.
Physical and Somatic Phenotypes: Multidimensional frailty status quantified using the Fried frailty criteria. Sarcopenia was evaluated as a latent construct driven by three synchronized observed metrics: skeletal muscle mass index (SMI), handgrip strength, and 6-m gait speed.
Psychological and Affective Phenotypes: Depressive severity and generalized anxiety levels evaluated via the 17-item Hamilton Depression Rating Scale (HAMD-17) and the 7-item Generalized Anxiety Disorder Scale (GAD-7), respectively.

A total of 321 eligible patients with complete baseline clinical and biomarker datasets were initially enrolled in the prospective observational cohort. Over the 5-year follow-up trajectory, a subset of patients experienced intermittent missingness, voluntary withdrawal, or attrition (with 35 participants lost to follow-up). Rather than simple listwise deletion which introduces severe sample truncation bias, longitudinal missing data patterns were robustly preserved and statistically addressed via Full Information Maximum Likelihood (FIML) for structural equation frameworks and multiple imputation by chained equations (MICE) for whole-cohort sensitivity checkups (15, 16), thereby ensuring the maximum available statistical power and internal validity of the 5-year longitudinal analysis.

### 2.4 Diagnostic adjudication for incident RLS

The prospective clinical diagnosis of incident RLS was rigorously determined in strict accordance with the consensus criteria established by the International Restless Legs Syndrome Study Group (IRLSSG) (17). To satisfy a formal diagnosis, a patient must consistently fulfill all four standard core essential criteria:

- An irresistible urge to move the legs, usually accompanied by, or felt to be caused by, uncomfortable and unpleasant sensations in the legs;
- The urge to move or unpleasant sensations begin or worsen during periods of rest or inactivity such as sitting or lying down;
- The urge to move or unpleasant sensations are partially or totally relieved by movement, such as walking or stretching, at least as long as the activity continues;
- The urge to move or unpleasant sensations are worse in the evening or night than during the day, or only occur in the evening or night.

To prevent diagnostic ambiguity and ensure methodological consistency, all participants underwent standardized, face-to-face clinical evaluations conducted annually by certified neurologists who were masked to the patients’ baseline phenotypic and biochemical profiles. The diagnostic workflow integrated structured patient-reported symptom inventories, detailed medical history, and comprehensive physical and neurological examinations. Alternative clinical mimics that could confound RLS diagnostics (such as positional discomfort, venous stasis, leg cramps, or peripheral vascular disease) were systematically excluded to guarantee maximum diagnostic specificity for incident RLS cases across the 5-year longitudinal follow-up.

### 2.5 Comprehensive evaluation and operationalization of risk factors

#### 2.5.1 Psychological and affective phenotypes

Psychological health indicators encompassed multidimensional assessments of depressive severity and generalized anxiety status. Depressive symptoms were evaluated utilizing the clinician-administered HAMD-17, where a total score > 7 clinically operationalized the presence of depressive distress (18). Generalized anxiety levels were quantified through the GAD-7, with a total score > 5 serving as the validated clinical threshold signifying the presence of anxiety symptoms (19).

#### 2.5.2 Physical and somatic phenotypes

Physical performance and somatic wasting matrices were operationalized through standardized phenotypic definitions:

- Multidimensional Frailty: Frailty was evaluated utilizing the phenotypic Fried frailty criteria, capturing five distinct clinical dimensions: unintentional weight loss, self-reported exhaustion/fatigue, objective decrease in handgrip strength, slow walking speed, and reduced physical activity. Each dimension contributed 1 point, yielding a total score ranging from 0 to 5, where a cumulative score ≥ 3 was rigorously classified as indicative of clinical frailty (20).
- Sarcopenia: Sarcopenia was evaluated and defined in strict accordance with the 2019 consensus criteria established by the Asian Working Group for Sarcopenia (AWGS 2019) (21). Objective muscle mass was quantified via the SMI, with low muscle mass defined as an SMI < 7 kg/m^2^ for males and < 5.7 kg/m for females. Objective muscle strength was measured via handgrip strength, with reduced muscle strength defined as < 28 kg for males and < 18 kg for females. Sarcopenia was defined as the synchronous presence of both low muscle mass and reduced muscle strength. Additionally, a 6-m gait speed assessment was performed to capture physical performance as part of the holistic latent trajectory specification.

#### 2.5.3 Laboratory biomarkers and clinical stratification

Venous blood samples were collected under fasting conditions using standardized laboratory protocols. To optimize statistical power and capture full-range predictive values, laboratory parameters—including serum albumin, hemoglobin, ferritin, CRP, serum calcium, serum phosphorus, and iPTH—were treated primarily as continuous variables within the longitudinal multivariable models. Concurrently, for baseline characterization and clinical stratification, these parameters were stratified using predefined, widely recognized clinical risk thresholds derived from established renal literature (22, 23): hemoglobin (≥ 10 vs. < 10 g/dL), ferritin (≥ 200 vs. < 200 ng/mL), serum albumin (≥ 3.5 vs. < 3.5 g/d), and CRP (≥ 6 vs. < 6 ng/mL).

### 2.6 Statistical analysis

*Baseline Standardization and Precise Matching Framework*: Continuous variables were presented as mean ± standard deviation (mean ± SD) or medians with interquartile ranges (IQRs) based on the Shapiro–Wilk normality test, while categorical variables were expressed as frequencies and percentages [*n* (%)]. Inter-group baseline differences were evaluated using the independent *t*-test, Mann–Whitney *U* test, Chi-square test, or Fisher’s exact test where appropriate. To rigorously control for demographic and clinical confounding factors at the baseline cross-section, a 1:1 clinical exact matching protocol was executed. Confounding background variables—including age, sex, BMI, dialysis vintage, vascular access type, dialysis adequacy (kt/V), CCI, and baseline treatments (ESA and iron supplement utilization)—were strictly homogenized. The post-matching covariate equilibrium was evaluated using the Standardized Mean Difference (SMD), with an SMD < 0.06 indicating an optimal balance.

*Longitudinal Association, Time-Dynamic Interaction, and Predictive Window Diagnostics*: To accommodate the repeated-measure property of time-varying clinical phenotypes and estimate their prospective predictive values for 5-year incident RLS, longitudinal GEE with logit link functions were constructed. Single-domain frameworks (Models 1–3) were sequentially integrated into a comprehensive, mutually adjusted Multidimensional Model (Model 4). Multiplicative interaction terms (e.g., Sarcopenia × Depression) were introduced within the GEE architecture to evaluate synergistic risk compounding effects. To capture the longitudinal trajectory variation, time-dynamic interaction analyses were performed from Year 1 through Year 5. High-order multiplicative interaction matrices (extending from two-way to four-way pan-phenotypic clustering) were constructed to quantify systemic cumulative risks. Furthermore, to address potential immortal time bias and verify early-screening efficiency, landmark analyses were executed by establishing fixed clinical milestones at Year 1 and Year 2 for participants remaining at risk.

*Methodological Robustness and Bias Diagnostics*: To evaluate the structural sensitivity of the primary GEE estimations against alternative model specifications, the longitudinal frameworks were systematically re-estimated using alternative working correlation structures, specifically first-order autoregressive [AR(1)] and exchangeable matrices. Outlier sensitivity checks were performed by re-running models after excluding extreme phenotypic values (defined as exceeding ± SD). To rigorously rule out residual unmeasured confounding or systematic artifactual bias, a negative control outcome analysis was performed using an identical multivariable GEE architecture against a theoretically and biologically unrelated clinical endpoint. Predictive discrimination capacities of individual phenotypes and the combined multi-system model were quantified using the Harrell’s Concordance Index (C-index).

*Latent Growth Curve Mediation Analysis*: To explore the longitudinal pathways and dual-channel mediation patterns (baseline cross-section vs. chronic deterioration rate), separate and decoupled LGCM were evaluated individually for each phenotype to prevent parameter overparameterization and ensure optimal parameter stability under the constraints of our matched cohort size (*N* = 192). Each independent model constrained the number of estimated parameters (*q*) between 12 and 18, strictly adhering to the recommended sample size-to-parameter ratio (N:*q* ≥ 10:1) for stable structural equation modeling estimation (24, 25). For each individual framework, cumulative longitudinal duration/dialysis exposure served as the independent variable (*X*), while the baseline level (Latent Intercept, *I*) and the longitudinal rate of change (Latent Slope, *S*) served as parallel mediators (*M*), and incident RLS served as the longitudinal categorical outcome (*Y*). Longitudinal measurement invariance for latent constructs—specifically sarcopenia, which was modeled as a latent construct driven by three observed markers (SMI, handgrip strength, and 6-m gait speed)—was rigorously validated across the 5 waves using confirmatory factor analysis (CFA), exhibiting invariant standardized factor loadings ranging from 0.70 to 0.78 (*p* < 0.001) (26).

Longitudinal missingness within the primary matched cohort was handled via Full Information Maximum Likelihood (FIML) to maximize statistical power (27). Within this structural equation modeling framework, the indirect mediation effects were quantified using the standard product-of-coefficients (*αβ*) approach. To address the inherent non-normality of product terms and guarantee maximum statistical robustness, the statistical significance and stability of direct, indirect, and total path coefficients (*β*) were validated using bias-corrected percentile bootstrapping with 5,000 resamples. Point estimates and asymmetric 95% confidence intervals (CIs) were reported, where an indirect effect was considered statistically significant if its bootstrap CI excluded zero (28). The proportion of the total effect mediated was quantified as the ratio of the indirect effect to the total effect (Proportion Mediated = Indirect Effect/Total Effect). As an additional methodological defense, robustness checks for path estimates were replicated within the full unmatched cohort (*N* = 321) utilizing MICE across 50 datasets (29).

### 2.7 Two-sample bidirectional and network mediation Mendelian randomization

#### 2.7.1 Data sources and instrumental variable selection

Publicly available GWAS summary statistics were leveraged to triangulate observational findings at the germline level (sources detailed in Supplementary Table S1). Genetic proxies were meticulously formulated to reflect specialized phenotypes: (1) Renal Function Profile: represented by eGFR and UACR to approximate the genetic risk of advanced renal impairment; (2) Sarcopenia: proxied by appendicular lean mass (ALM) (30); and (3) Dopamine Metabolism: proxied by plasma dopamine 3-O-sulfate levels (31). Validated GWAS datasets for depression, anxiety, and the frailty index (FI) were utilized without proxy modifications.

To satisfy the three core MR assumptions (relevance, independence, and exclusivity), genetic instrumental variables were extracted under genome-wide significance (*p* < 5 × 10^−8^). Strict linkage disequilibrium clumping was executed (*r^2^* < 0.001 within a 10,000-kb window) using the European 1,000 Genomes reference panel to guarantee instrument independence. Weak instrument bias was ruled out by restricting selection to IVs with an *F*-statistic > 10 (*F = β^2^/SE^2^*). Loci associated with potential multi-phenotypic confounding were manually cross-checked and pruned via the GWAS Catalog (32).

#### 2.7.2 Network mediation architecture and causal diagnostics

A two-step mediation MR framework was constructed to delineate the genetically predicted causal cascade from renal dysfunction to downstream dopamine metabolism. Step 1: Two-sample MR evaluated the causal effects of renal exposures (eGFR, UACR) on individual potential mediators (ALM, depression, anxiety, FI) and the final metabolic outcome (*β_1_*). Step 2: Significant intermediate phenotypes from Step 1 were operationalized as exposures against dopamine 3-O-sulfate to estimate the secondary path effects (*β_2_*). The indirect mediation effect was quantified using the product-of-coefficients method (*β*_indirect_ = *β_1_* × *β_2_*), with its asymmetric standard error and 95% CIs derived via the Delta method (33). The proportion mediated was defined as *β*_indirec_/*β_total_*.

The inverse variance weighted (IVW) method served as the primary estimator to synthesize Wald ratios across instruments. Robustness and directional consistency were cross-validated using MR-Egger, Weighted Median, Simple Mode, and Weighted Mode methods. Horizontal pleiotropy was diagnosed using the MR-Egger intercept test, and instrumental heterogeneity was quantified using Cochran’s Q statistic (*p* < 0.05 indicating significant violations). Potential small-sample bias and single-variant driving effects were visually inspected via funnel plots and leave-one-out sensitivity analyses. All genetic analyses were performed using the TwoSampleMR package in R (version 4.5.2) with statistical significance set at a two-tailed *p* < 0.05.

## 3 Results

### 3.1 Baseline characteristics of the study cohort

The baseline characteristics of the study population (*N* = 321) before and after 1:1 demographics and clinical matching are summarized in Table 1. In the overall unmatched cohort, patients who subsequently developed incident RLS during the 5-year follow-up exhibited higher crude rates of baseline hypertension (51.0% vs. 38.2%, *p* = 0.033) compared to the control group.

**Table 1:** Baseline characteristics of patients before and after demographics and clinical matching stratified by 5-year incident RLS status.

Characteristic	Before matching			After matching			
	Control (*n* = 225)	Incident RLS (*n* = 96)	*p* value	Control (*n* = 96)	Incident RLS (n = 96)	*p* value	SMD
Demographics and matched clinical background							
Age (years)	63.00 ± 5.07	64.46 ± 5.36	0.069	64.12 ± 5.15	64.46 ± 5.36	0.652	0.06
Male, *n* (%)	124 (55.1%)	50 (52.1%)	0.883	51 (53.1%)	50 (52.1%)	0.885	0.02
BMI (kg/m^2^)	24.90 ± 1.74	24.80 ± 1.97	0.912	24.85 ± 1.76	24.80 ± 1.97	0.854	0.03
Dialysis vintage (months)	38.50 ± 12.40	41.20 ± 14.10	0.092	40.80 ± 13.20	41.20 ± 14.10	0.841	0.03
AVF, *n* (%)	198 (88.0%)	81 (84.4%)	0.385	82 (85.4%)	81 (84.4%)	0.848	0.03
Dialysis adequacy (Kt/V)	1.38 ± 0.15	1.35 ± 0.18	0.125	1.36 ± 0.16	1.35 ± 0.18	0.684	0.06
CCI	4.12 ± 1.25	4.38 ± 1.40	0.108	4.32 ± 1.31	4.38 ± 1.40	0.758	0.04
Baseline ESA use, *n* (%)	162 (72.0%)	74 (77.1%)	0.342	73 (76.0%)	74 (77.1%)	0.862	0.03
Baseline Iron supplements use, *n* (%)	140 (62.2%)	65 (67.7%)	0.351	64 (66.7%)	65 (67.7%)	0.881	0.02
Exposures, main phenotypes and biomarkers							
Serum albumin (g/dL)	3.84 ± 0.21	3.38 ± 0.23	<0.001	3.74 ± 0.22	3.38 ± 0.23	<0.001	1.60
Hemoglobin (g/dL)	11.12 ± 0.66	9.99 ± 0.63	<0.001	10.92 ± 0.70	9.99 ± 0.63	<0.001	1.40
Ferritin (ng/mL)	323.15 ± 77.70	230.35 ± 47.14	<0.001	319.50 ± 70.80	230.35 ± 47.14	<0.001	1.48
CRP (mg/L)	5.49 ± 1.09	10.01 ± 1.36	<0.001	5.65 ± 1.20	10.01 ± 1.36	<0.001	3.40
Serum calcium (mg/dL)	9.12 ± 0.45	8.75 ± 0.52	<0.001	9.08 ± 0.48	8.75 ± 0.52	<0.001	0.66
Serum phosphorus (mg/dL)	4.85 ± 0.92	5.42 ± 1.15	<0.001	4.92 ± 0.98	5.42 ± 1.15	<0.001	0.47
iPTH (pg/mL)	245.5 ± 82.0	312.4 ± 95.5	<0.001	252.0 ± 84.5	312.4 ± 95.5	<0.001	0.67
Sarcopenia, *n* (%)	12 (5.3%)	47 (49.0%)	<0.001	7 (7.3%)	47 (49.0%)	<0.001	1.05
Fried score	1.44 ± 1.08	3.98 ± 0.75	<0.001	1.85 ± 1.02	3.98 ± 0.75	<0.001	2.37
HAMD-17 score	7.10 ± 1.46	17.64 ± 2.56	<0.001	7.38 ± 1.50	17.64 ± 2.56	<0.001	5.01
GAD-7 score	2.48 ± 1.09	7.68 ± 1.15	<0.001	2.25 ± 1.05	7.68 ± 1.15	<0.001	4.90

Data are expressed as mean ± standard deviation for continuous variables, and as count (percentage) for categorical variables. (A) Statistical significance was evaluated using the independent *t*-test or Mann–Whitney *U* test for continuous data, and the Chi-square test or Fisher’s exact test for categorical data. (B) 1:1 clinical exact matching was performed based on baseline demographics and clinical backgrounds (from Age through Baseline Iron supplements use) to minimize potential confounding. A SMD < 0.10 indicates an optimal balance between the control and incident RLS groups post-matching. AVF, arteriovenous fistula; BMI, body mass index; CCI, Charlson Comorbidity Index; CRP, C-reactive protein; ESA, erythropoiesis-stimulating agent; GAD-7, 7-item Generalized Anxiety Disorder scale; HAMD-17, 17-item Hamilton Depression Rating Scale; iPTH, intact parathyroid hormone; RLS, restless legs syndrome; SMD, standardized mean difference.

Following the execution of the optimal multivariate matching algorithm based on 96 incident RLS cases and 96 matched controls, a high level of covariate balance was achieved. Fundamental demographics and dialysis infrastructure parameters—including age, sex, BMI, dialysis vintage, vascular access type (AVF percentage), dialysis adequacy (Kt/V), and CCI—demonstrated substantial numerical alignment between the two sub-cohorts (all *p* > 0.033, SMD < 0.06). Concurrently, no statistically significant differences were observed in the baseline utilization of ESA or iron supplements (*p* = 0.862 and *p* = 0.881, respectively), suggesting that the foundational clinical and treatment backgrounds were adequately homogenized.

In contrast, substantial phenotypic and biochemical divergence persisted between the matched groups (all *p* < 0.001). Patients in the incident RLS group presented with lower baseline serum albumin (3.38 ± 0.23 vs. 3.74 ± 0.22 g/dL), hemoglobin (9.99 ± 0.63 vs. 10.92 ± 0.70 g/dL), and ferritin levels, alongside markedly elevated CRP concentrations (10.01 ± 1.36 vs. 5.56 ± 1.20 mg/dL). Furthermore, severe disruptions in bone and mineral metabolism were evident in the RLS group, characterized by suppressed serum calcium, elevated serum phosphorus, and heightened iPTH levels. Notably, the baseline prevalence of sarcopenia (49.0% vs. 7.3%) and the severity of multi-dimensional frailty and psychological distress—evidenced by significantly higher Fried frailty criteria, HAMD-17, and GAD-7 scores—were disproportionately concentrated in patients who subsequently transitioned to incident RLS. These baseline imbalances underscore a strong prospective clustering of systemic wasting, mineral disorders, and somatopsychic vulnerability preceding clinical RLS manifestation.

### 3.2 Longitudinal associations, time-dynamic interventions, and multi-way interaction effects

The longitudinal associations between time-varying clinical phenotypes and incident RLS were evaluated using GEE models (Table 2).

**Table 2:** Longitudinal associations of biochemical, physical, and psychological phenotypes with incident RLS based on GEE models.

Variable	Univariable OR (95% CI)	*P* value	Multivariable a OR (95% CI)	*P* value
Model 1. Biochemical and mineral metabolism model				
Serum albumin (g/dL)	0.71 (0.60–0.84)	<0.001	0.79 (0.65–0.95)	0.013
Hemoglobin (g/dL)	0.82 (0.74–0.91)	<0.001	0.89 (0.78–1.01)	0.074
Ferritin (ng/mL)	0.93 (0.88–0.98)	0.009	0.96 (0.90–1.02)	0.181
CRP (mg/L)	1.24 (1.16–1.32)	<0.001	1.18 (1.10–1.27)	<0.001
Serum calcium (mg/dL)	0.81 (0.70–0.94)	0.006	0.87 (0.74–1.03)	0.108
Serum phosphorus (mg/dL)	1.19 (1.08–1.31)	<0.001	1.13 (1.01–1.26)	0.028
iPTH (pg/mL)	1.07 (1.02–1.12)	0.004	1.05 (1.00–1.10)	0.047
Model 2. Physical phenotype model				
Sarcopenia	2.18 (1.71–2.79)	<0.001	1.74 (1.33–2.28)	<0.001
Fried score	1.42 (1.28–1.57)	<0.001	1.31 (1.17–1.47)	<0.001
Model 3. Psychological phenotype model				
HAMD-17 score	1.15 (1.10–1.21)	<0.001	1.12 (1.06–1.18)	<0.001
GAD-7 score	1.17 (1.11–1.23)	<0.001	1.14 (1.08–1.21)	<0.001
Model 4. Integrated multidimensional model				
Serum albumin (g/dL)	—	—	0.84 (0.69–1.02)	0.078
CRP (mg/L)	—	—	1.04 (0.861–1.25)	0.70
Sarcopenia	—	—	1.58 (1.18–2.11)	0.002
HAMD-17 score	—	—	1.10 (1.04–1.17)	0.001
Sarcopenia × Depression	—	—	1.50 (1.10–2.05)	0.012

Data are presented as ORs with 95% CIs, estimated using longitudinal GEE models. All multivariable models (Models 1–4) were adjusted for prespecified clinically relevant baseline covariates, including age, sex, BMI, dialysis vintage, vascular access type, dialysis adequacy (Kt/V), and CCI, to minimize potential confounding. Model 1 evaluated biochemical and inflammatory parameters; Model 2 evaluated physical functional parameters; and Model 3 evaluated psychological parameters. The corresponding univariable estimates for each parameter were independently calculated and are presented within their respective single-domain models. In Model 4, the four target phenotypes (depression, anxiety, frailty, and sarcopenia) were simultaneously entered into an integrated multidimensional regression framework to assess their mutually independent predictive effects. BMI, body mass index; CCI, Charlson Comorbidity Index; CRP, C-reactive protein; GAD-7, 7-item Generalized Anxiety Disorder scale; HAMD-17, 17-item Hamilton Depression Rating Scale; iPTH, intact parathyroid hormone; Kt/V, dialysis adequacy index.

In single-domain multivariable analyses adjusting for prespecified baseline covariates, statistically significant independent predictors emerged across multiple domains. In Model 1 (biochemical and mineral metabolism), lower serum albumin (Adjusted OR = 0.79, *p* = 0.013), elevated CRP (Adjusted OR = 1.18, *p* < 0.001), higher serum phosphorus (Adjusted OR = 1.13, *p* = 0.028), and higher iPTH (Adjusted OR = 1.05, *p* = 0.047) were associated with an increased risk of RLS. Hemoglobin, serum calcium, and ferritin did not exhibit significant associations (*p* > 0.005). For physical phenotypes (Model 2), both sarcopenia (Adjusted OR = 1.74, *p* < 0.001) and higher Fried scores (Adjusted OR = 1.31, *p* < 0.001) independently predicted incident RLS. For psychological phenotypes (Model 3), higher HAMD-17 (Adjusted OR = 1.12, *p* < 0.001) and GAD-7 scores (Adjusted OR = 1.14, *p* < 0.001) were positively correlated with RLS development.

In the Integrated Multidimensional Model (Model 4), sarcopenia (Adjusted OR = 1.58, 95% CI = 1.18 to 2.11, *p* = 0.002), and HAMD-17 score (Adjusted OR = 1.10, 95% CI = 1.04 to 1.17, *p* < 0.001) maintained robust, mutually independent predictive values. Serum albumin did not retain statistical significance (Adjusted OR = 0.84, *p* = 0.078). Notably, a significant multiplicative Sarcopenia depression interaction was identified within Model 4 (Adjusted OR = 1.50, 95% CI = 1.10 to 2.05, p = 0.0212), suggesting a synergistic risk compounding effect between musculoskeletal depletion and depressive severity.

**Figure 2:**
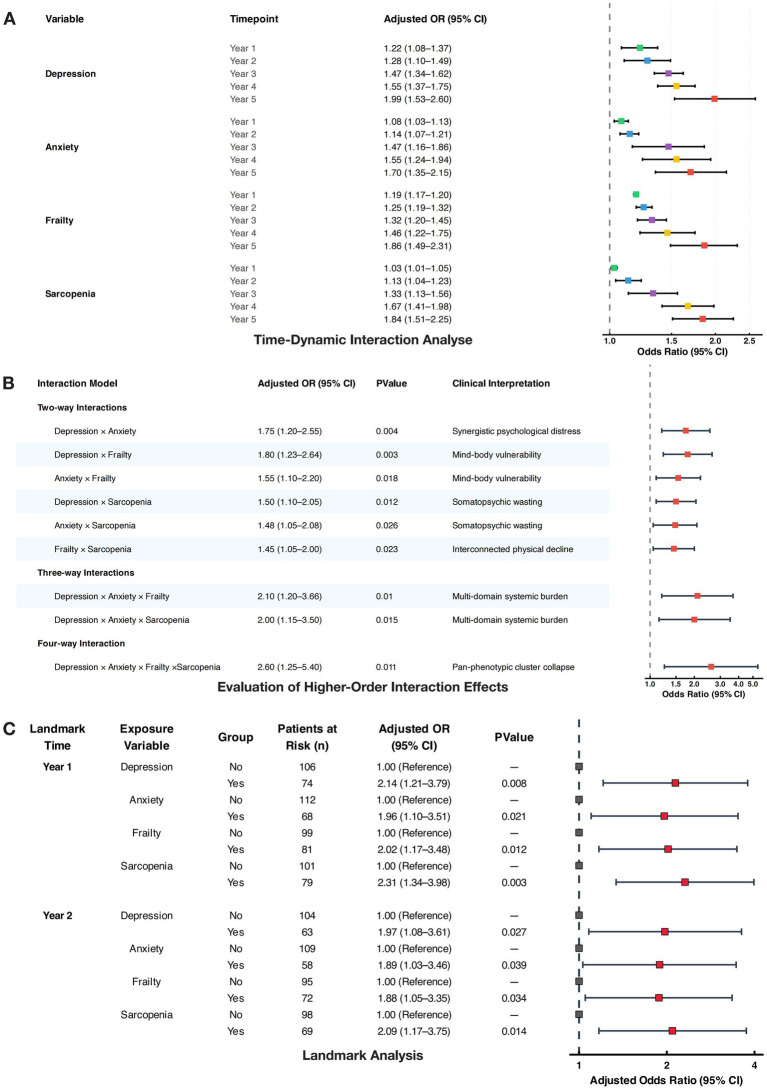
Time-dynamic profiles, higher-order interactions, and landmark analyses of the studied clinical phenotypes. Horizontal bars represent 95% CIs. All multi-variable models are fully adjusted for pre-specified baseline covariates, including age, sex, BMI, dialysis vintage, vascular access type, dialysis adequacy (Kt/V), and CCI. **(A)** Time-Dynamic Interaction Analyse: Longitudinal GEE assessments showing changes in risk magnitudes (Adjusted OR, 95% CI) for depression, anxiety, frailty, and sarcopenia sequentially from Year 1 to Year 5. **(B)** Evaluation of Higher-Order Interaction Effects: Multivariable-adjusted product-term analysis displaying multiplicative interactions among the target clinical dimensions, alongside corresponding clinical interpretations. **(C)** Landmark Analysis: Risk distributions and adjusted ORs for the exposure variables evaluated at specific clinical landmark intervals (Year 1 and Year 2). CI, confidence interval; GEE, generalized estimating equation; OR, odds ratio.

Furthermore, time-dynamic interaction analyses (Figure 2A) indicated a progressive intensification of these prospective associations over the 5-year follow-up period. The adjusted OR for sarcopenia increased from 1.03 (95% CI = 1.01 to 1.05) in Year 1 to 1.85 (95% CI = 1.51 to 2.25) in Year 5, while depression concurrently rose from an OR of 1.22 to 1.99, indicating a cumulative systemic burden over time.

Additionally, evaluation of higher-order interaction effects (Figure 2B) demonstrated extensive cross-talk among these phenotypes. Beyond the significant two-way and three-way interaction structures, the concurrent presence of all four aberrant conditions—manifesting as a pan-phenotypic clustering of depression, anxiety, frailty, and sarcopenia—was associated with a pronounced risk surge for incident RLS. This clustering presented a distinct four-way multiplicative interaction effect (Adjusted OR = 2.60, 95% CI = 1.25 to 5.40, *p* = 0.011), highlighting a substantial, compounding risk acceleration for subsequent RLS onset.

### 3.3 Clinical prediction window verification via landmark analysis

To address potential immortal time bias and evaluate the clinical utility of these findings within specific follow-up intervals, a rigorous landmark analysis was performed (Figure 2C). Fixed at the 1-year landmark timepoint (patients remaining at risk = 180), participants categorized with sarcopenia, frailty, depression, or anxiety at that specific milestone exhibited a significantly higher cumulative incidence of RLS during the subsequent follow-up interval compared to their unaffected counterparts. This predictive capacity remained robust at the 2-year landmark milestone (patients remaining at risk = 167), where pre-existing sarcopenia (adjusted OR = 2.09, 95% CI: 1.17–3.75, *p* = 0.014) and depression (adjusted OR = 1.97, 95% CI: 1.08–3.61, *p* = 0.027) continued to serve as potent, independent long-term predictors of subsequent RLS onset, thereby supporting the clinical stability and early-screening value of these multi-system phenotypes.

### 3.4 Sensitivity and systematic bias analyses

To evaluate the robustness of the primary multivariable GEE estimations, sensitivity analyses were performed by re-estimating the models across alternative working correlation structures—including autoregressive [AR(1)] and exchangeable specifications—and by excluding extreme outliers (Table 3). The estimated effects of key risk factors on RLS risk remained consistently stable across these four specifications. Specifically, the adjusted ORs ranged from 1.83 to 1.86 for depression, 1.45 to 1.48 for anxiety, 1.61 to 1.64 for frailty, and 1.40 to 1.42 for sarcopenia, with all associations maintaining statistical significance. These diagnostics indicate that the primary findings were not sensitive to correlation structure configurations or extreme value distributions, supporting the stability of our main conclusions.

**Table 3:** Robustness analysis of multivariable GEE results.

Characteristic variable	Basic model OR (95% CI)	AR(1) working correlation OR (95% CI)	Exchangeable working correlation OR (95% CI)	Excluding outliers OR (95% CI)
Depression	1.85 (1.32–2.60)	1.84 (1.31–2.58)	1.86 (1.33–2.61)	1.83 (1.30–2.57)
Anxiety	1.47 (1.05–2.05)	1.46 (1.04–2.04)	1.48 (1.06–2.06)	1.45 (1.03–2.03)
Frailty	1.63 (1.18–2.26)	1.62 (1.17–2.25)	1.64 (1.19–2.27)	1.61 (1.16–2.24)
Sarcopenia	1.41 (1.01–1.96)	1.42 (1.02–1.97)	1.41 (1.01–1.96)	1.40 (1.00–1.95)

Data are presented as multivariable-adjusted ORs with corresponding 95% CIs, estimated using longitudinal GEE models. *Model configurations*: The Basic Model represents the primary multivariable-adjusted longitudinal framework incorporating the independent core phenotypes and prespecified baseline covariates, including age, sex, BMI, dialysis vintage, vascular access type, dialysis adequacy (Kt/V), and Charlson Comorbidity Index, to minimize potential confounding. To assess structural sensitivity, the GEE models were sequentially re-estimated using AR(1) and exchangeable working correlation structures. “Excluding Outliers” refers to the sensitivity analysis performed after removal of extreme values, defined as observations exceeding 3 standard deviations from the corresponding phenotypic distribution means. Robustness Evaluation: The consistent directionality and comparable magnitudes of the estimated ORs across different working correlation structures and outlier exclusion frameworks support the statistical robustness and structural stability of the identified longitudinal phenotypic associations with incident RLS. AR(1), first-order autoregressive; BMI, body mass index; CI, confidence interval; GEE, generalized estimating equations; OR, odds ratio; RLS, restless legs syndrome.

To address potential systematic bias or residual confounding, a negative control outcome theoretically unrelated to the primary exposures was introduced using an identical multivariable GEE architecture (Supplementary Table S2). No significant associations were observed between the negative control and any primary exposures, including sarcopenia (OR = 1.05, 95% CI: 0.89–1.24, *p* = 0.58) and severe depression (OR = 1.08, 95% CI: 0.92–1.27, *p* = 0.35). These results suggest that the investigated physical and psychological phenotypes do not erroneously predict a biologically irrelevant outcome, reducing the likelihood of systemic confounding.

In conclusion, these multi-model sensitivity checks and negative control validations consistently aligned with our primary analyses, enhancing the internal validity and reliability of the identified risk axes connecting depression, anxiety, frailty, sarcopenia, and incident RLS.

### 3.5 Longitudinal mediation patterns of phenotypic trajectories based on latent growth curve models

To explore the longitudinal pathways through which physical and psychological phenotypes may contribute to neurological vulnerability, four separate latent growth curve mediation models were evaluated individually (Table 4). All decoupled models demonstrated optimal global fit criteria (CFI ≥ 0.959, RMSEA ≤ 0.048, SRMR ≤ 0.038), confirming high structural stability under sample size constraints.

**Table 4:** Latent growth curve model analysis of the direct and indirect mediating effects of depression, anxiety, frailty, and sarcopenia on RLS risk in maintenance hemodialysis patients.

Mediator	Latent intercept β (SE)	Latent slope β (SE)	Direct effect of MHD on RLS β (SE)	Indirect effect β (SE)	Total effect β (SE)	*P*
Depression	0.21 (0.05)	0.18 (0.04)	0.15 (0.03)	0.12 (0.02)	0.27 (0.03)	<0.001
Anxiety	0.17 (0.04)	0.14 (0.03)	0.12 (0.03)	0.10 (0.02)	0.22 (0.03)	<0.001
Frailty	0.19 (0.05)	0.16 (0.04)	0.13 (0.03)	0.11 (0.02)	0.24 (0.03)	<0.001
Sarcopenia	0.22 (0.06)	0.18 (0.04)	0.14 (0.03)	0.12 (0.03)	0.26 (0.03)	<0.001

*Model architecture*: To prevent parameter overparameterization and ensure statistical robustness under sample size constraints, four separate and independent latent growth curve mediation models were constructed for each individual phenotypic trajectory rather than an all-inclusive simultaneous model. For each individual model, cumulative longitudinal duration/dialysis exposure served as the independent variable (X); the baseline level (Latent Intercept) and the rate of change (Latent Slope) of each phenotype served as parallel mediators (M); and incident RLS served as the longitudinal categorical outcome (Y). Sarcopenia was modeled as a latent construct driven by three observed markers: SMI, handgrip strength, and 6-m gait speed. Longitudinal measurement invariance was established across the 5 waves, with CFA exhibiting invariant standardized factor loadings ranging from 0.70 to 0.78 (*P* < 0.001). Statistical estimation: Longitudinal missingness was handled via FIML. The significance of both direct and indirect mediating effects was validated using bias-corrected percentile bootstrapping with 5,000 resamples to guarantee maximum stability. *Model fit diagnostics*: Each independent model demonstrated optimal global fit criteria: (1) Depression Model: CFI = 0.962, RMSEA = 0.045, SRMR = 0.035; (2) Anxiety Model: CFI = 0.968, RMSEA = 0.040, SRMR = 0.032; (3) Frailty Model: CFI = 0.959, RMSEA = 0.048, SRMR = 0.038; (4) Sarcopenia Model: CFI = 0.960, RMSEA = 0.045, SRMR = 0.035. β, standardized path coefficient; CFA, confirmatory factor analysis; CFI, comparative fit index; MHD, maintenance hemodialysis; RLS, restless legs syndrome; RMSEA, root mean square error of approximation; SE, standard error; SMI, skeletal muscle mass index; SRMR, standardized root mean square residual.

The structural decomposition revealed that both the initial baseline severity (Latent Intercept) and the longitudinal rate of change (Latent Slope) of the investigated phenotypes functioned as significant parallel mediators tracking incident RLS risk. Specifically, cumulative longitudinal dialysis exposure exerted a significant direct effect (*β* = 0.15, SE = 0.03) alongside a potent indirect mediating effect (*β* = 0.12, SE = 0.02) driven by the depression trajectory, culminating in a total effect of *β* = 0.27 (SE = 0.03, *p* < 0.001; accounting for 44.4% of the total effect). Similarly, the longitudinal trajectory of anxiety mediated the risk pathway with an indirect effect of *β* = 0.10 (SE = 0.02, Proportion Mediated = 45.5%; total effect *β* = 0.22, *p* < 0.001).

Parallel potencies were observed within the physical wasting phenotypes. Frailty and sarcopenia trajectories yielded highly significant indirect mediating effects of *β* = 0.11 and *β* = 0.12, respectively, which translated to substantial mediation proportions of 45.8 and 46.2% of their cumulative risk pathways (both total effects *p* < 0.001). Collectively, these findings support the presence of dual-channel longitudinal mediation patterns, suggesting that these multi-system impairments may longitudinally contribute to increased RLS susceptibility not only through their baseline clinical cross-section but also via their chronic, time-dependent deterioration during follow-up.

### 3.6 Full-population and multiple imputation-based sensitivity analyses for LGCM path estimates

To evaluate the statistical stability of the latent growth curve mediation pathways against sample configurations and missingness assumptions, a full-population sensitivity analysis was performed (Supplementary Table S3). By reverting to the full, unmatched cohort (*N* = 321) and utilizing multiple imputation (MI) across 50 datasets to interpolate longitudinal missingness, all four independent structural models demonstrated acceptable global fit criteria (CFI ≥ 0.952, RMSEA ≤ 0.052, SRMR ≤ 0.041) under full multivariate covariate adjustment.

Within this maximum available data configuration, the standardized indirect path coefficients (*β*) exhibited substantial numerical and directional invariance compared to the primary matched-cohort models. Specifically, significant indirect mediating effects persisted for depression (*β* = 0.10, SE = 0.02, Proportion Mediated = 41.7%; total effect *β* = 0.24, *p* < 0.001) and anxiety (*β* = 0.09, SE = 0.02, Proportion Mediated = 45.0%; total effect *β* = 0.20, *p* < 0.001). Parallel robust mediation channels were maintained for the physical wasting phenotypes, with substantial indirect effects identified for frailty (*β* = 0.10, SE = 0.02, Proportion Mediated = 45.5%) and sarcopenia (*β* = 0.11, SE = 0.03, Proportion Mediated = 45.8%; both total effects *p* < 0.001). These multi-model replications suggest that the identified somatopsychic-neurological mediation channels are structurally stable and unlikely to be artifactual products of sample selection or specific missing data algorithms, thereby minimizing concerns regarding model overparameterization.

### 3.7 Discrimination analysis of individual risk factors and the combined model

In univariable predictive analyses, the discriminative capacity of individual risk factors for RLS demonstrated variability. Depression was associated with the lowest predictive discrimination, evidenced by a C-index of 0.75 (95% CI: 0.71–0.79, *p* = 0.002), followed by frailty, which yielded a C-index of 0.78 (95% CI: 0.74–0.82, *p* = 0.015). In contrast, anxiety (C-index = 0.80, 95% CI: 0.76–0.84, *p* = 0.008) and sarcopenia (C-index = 0.82, 95% CI: 0.78–0.86, *p* = 0.020) exhibited comparatively higher discriminative abilities. The integration of these four risk factors into a multivariable combined prediction model resulted in a substantial enhancement of overall discriminative performance, achieving a C-index of 0.88 (95% CI: 0.83–0.93, *p* < 0.001). This finding underscores the superior predictive accuracy of the combined model in identifying patients at risk for developing RLS.

### 3.8 Causal effects of kidney function on depression, anxiety, and dopamine metabolism

**Figure 3:**
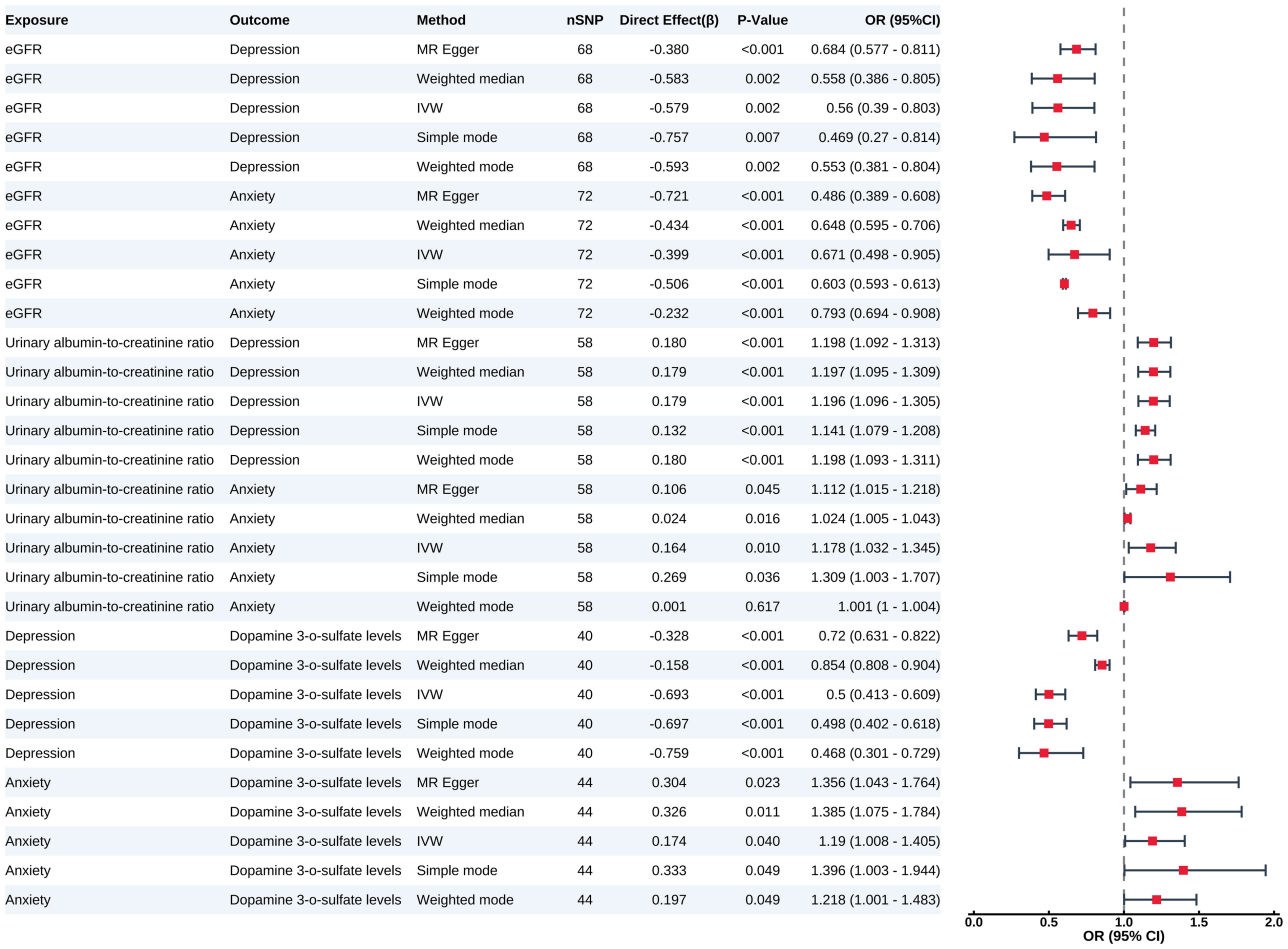
Mendelian randomization analysis for kidney function, affective traits, and dopamine metabolites. The forest plot presents the two sample MR estimates obtained using five analytical methods: MR-Egger, weighted median, IVW, simple mode, and weighted mode. Direct effect (β) represents the estimated causal effect size, from which the corresponding ORs and 95% CIs were derived. The vertical dashed line at OR = 1.0 indicates the null effect. nSNP denotes the number of single-nucleotide polymorphisms included in each analysis. Genetically predicted eGFR is inversely associated with depression and anxiety. Genetically predicted UACR shows positive causal links to both depression and anxiety. Genetically predicted depression is causally linked to decreased dopamine 3-osulfate levels, whereas anxiety shows a positive causal relationship with this metabolite. CI, confidence interval; eGFR, estimated glomerular filtration rate; IVW, inverse variance weighted; OR, odds ratio; UACR, urinary albumin-to-creatinine ratio.

All instrumental variables utilized in the MR analysis satisfied rigorous quality control metrics with statistics exceeding 10, indicating a minimal likelihood of weak instrument bias (Figure 3).

Two-sample MR analyses revealed significant causal links between genetically predicted kidney function and mental health outcomes (Figure 3). Specifically, a genetic predisposition to lower eGFR was associated with an increased risk of depression (IVW: *β* = −0.579, OR = 0.56, 95% CI = 0.390–0.803, *p* = 0.004) and anxiety (IVW: *β* = −0.399, OR = 0.671, 95% CI = 0.498–0.905, *p* < 0.001). Conversely, higher UACR was positively associated with both depression (IVW: *β* = 0.179, OR = 1.196, 95% CI = 1.096–1.305, *p* < 0.001) and anxiety (IVW: *β* = 0.164, OR = 1.178, 95% CI = 1.032–1.345, *p* = 0.01), suggesting that deteriorating renal function may causally elevate the susceptibility to adverse psychological outcomes.

Downstream analyses indicated that genetically predicted depression exerted a negative impact on dopamine 3-O-sulfate levels (IVW: *β* = −0.693, OR = 0.50, 95% CI = 0.413–0.609, *p* < 0.001), suggesting an association between heightened depressive symptoms and diminished dopamine metabolism. In contrast, anxiety demonstrated a marginal positive association with dopamine 3-O-sulfate (IVW: *β* = 0.174, OR = 1.19, 95% CI = 1.008–1.405, *p* = 0.04). Notably, neither eGFR nor UACR exhibited a direct causal association with dopamine 3-O-sulfate, ALM, or the FI (all *p* > 0.05; (Supplementary Table S2).

**Figure 4:**
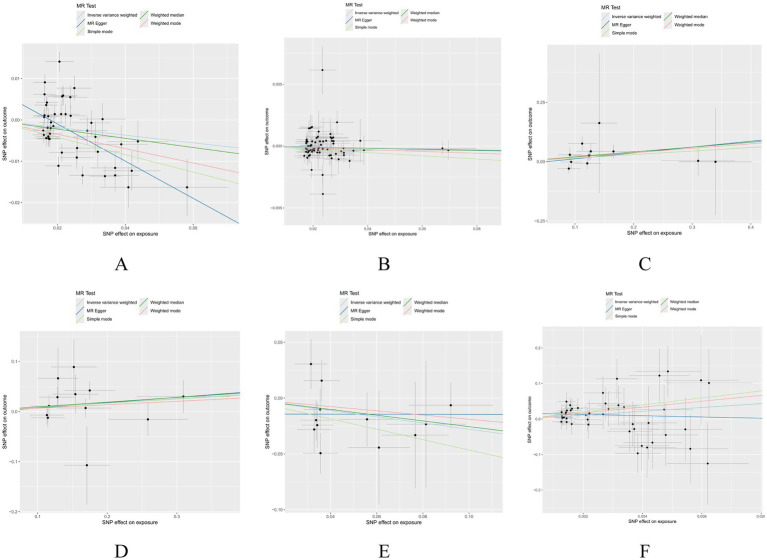
Two-step mediation Mendelian randomization analyses delineating the genetic pathway from kidney dysfunction to dopaminergic metabolism via psychological traits. Scatter plots depict genetic causal estimates derived from two-step mediation MR analyses designed to evaluate whether psychological traits mediate the relationship between kidney function and dopaminergic metabolism. Each point represents the SNP-specific association with the exposure (x-axis) and the corresponding association with the outcome (y-axis). Solid lines indicate causal estimates obtained from complementary MR approaches, IVW, MR-Egger regression, weighted median, simple mode, and weighted mode methods, enhancing robustness to horizontal pleiotropy and heterogeneity. Step 1 (exposure → mediator): **(A)** Genetically predicted eGFR and depression; **(B)** Genetically predicted eGFR and anxiety; **(C)** Genetically predicted UACR and depression; **(D)** Genetically predicted UACR and anxiety. Step 2 (mediator → outcome): **(E)** Genetically predicted depression and Dopamine 3-O-sulfate levels; **(F)** Genetically predicted anxiety and Dopamine 3-O-sulfate levels. Collectively, these analyses delineate a putative genetic mediation pathway in which renal dysfunction may influence dopaminergic metabolic alterations through affective disturbances. eGFR, estimated glomerular filtration rate; UACR, urinary albumin-to-creatinine ratio.

**Figure 5:**
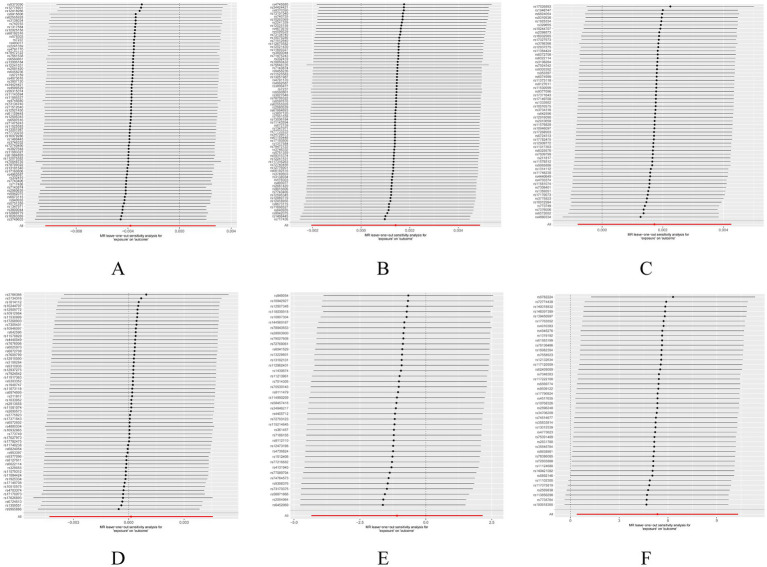
Forest plots of SNP-specific causal estimates in two-step mediation Mendelian randomization analyses. Forest plots displaying SNP-specific causal effect estimates derived from two-step mediation MR analyses. Each horizontal line represents the effect size and corresponding 95% confidence interval for an individual SNP using the Wald ratio method. The vertical dashed line indicates the null effect. These analyses allow visualization of heterogeneity across instrumental variables and assessment of the consistency of SNP-level effects contributing to the overall MR estimate. Step 1 (exposure → mediator): **(A)** eGFR on depression; **(B)** eGFR on anxiety; **(C)** UACR on depression; **(D)** UACR on anxiety. Step 2 (mediator → outcome): **(E)** Depression on Dopamine 3-O-sulfate levels; **(F)** Anxiety on Dopamine 3-O-sulfate levels. Collectively, these SNP-level estimates support the robustness of the proposed mediation pathway linking renal dysfunction, affective traits, and dopaminergic metabolism. eGFR, estimated glomerular filtration rate; UACR, urinary albumin-to-creatinine ratio.

Sensitivity analyses using MR-Egger, weighted median, simple mode, and weighted mode methods yielded directionally consistent effect estimates (Figure 3). Symmetrical distributions of SNP causal estimates in scatter plots (Figures 4A–F; Supplementary Figures 1A–H) supported the robustness of these findings. Furthermore, formal tests detected no significant horizontal pleiotropy or heterogeneity (Supplementary Table S4), while leave-one-out and funnel plots indicated stable causal estimations (Figures 5A–F; Supplementary Figures 2A–H, 3A–H).

**Figure 6:**
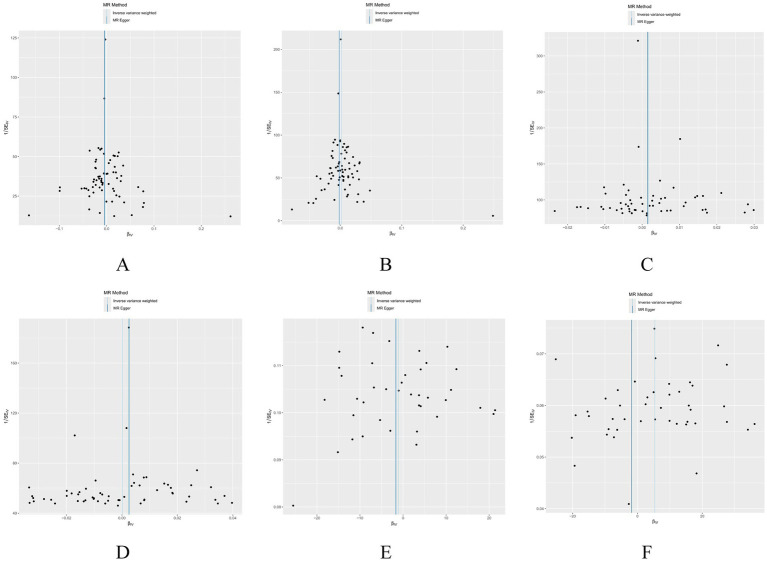
Funnel plots assessing horizontal pleiotropy and small-study effects in two-step mediation Mendelian randomization analyses. Funnel plots illustrating the distribution of SNP-specific causal estimates against their precision in two-step mediation MR analyses. Each dot represents an individual SNP. Symmetry around the vertical line (overall causal estimate) indicates the absence of directional horizontal pleiotropy or small-study effects. The vertical lines represent the pooled causal estimates derived from IVW and MR-Egger methods. Step 1 (exposure → mediator): **(A)** eGFR on depression; **(B)** eGFR on anxiety; **(C)** UACR on depression; **(D)** UACR on anxiety. Step 2 (mediator → outcome): **(E)** Depression on Dopamine 3-O-sulfate levels; **(F)** Anxiety on Dopamine 3-O-sulfate levels. Visual inspection of funnel plot symmetry supports the robustness of the instrumental variables and reduces the likelihood that the observed mediation pathway is driven by directional pleiotropy. eGFR, estimated glomerular filtration rate; UACR, urinary albumin-to-creatinine ratio.

### 3.9 Mediation analysis

Network MR mediation analysis demonstrated that the causal effects of renal impairment on dopamine metabolism were partially and directionally mediated by psychological health indicators (Figure 3).

Depression functioned as a prominent mediator within the renal-dopaminergic axes. In the pathway from eGFR to dopamine 3-O-sulfate via depression, the indirect effect was estimated at 0.401 (95% CI = 0.127–0.675, *p* = 0.004), accounting for approximately 41% of the total effect; genetically predicted lower eGFR linked to increased depression risk, which subsequently associated with suppressed dopamine metabolism. Similarly, in the UACR-to-dopamine pathway, depression mediated an indirect effect of −0.124 (95% CI: −0.194 to −0.054, *p* = 0.001), representing approximately 15% of the total effect.

Conversely, anxiety did not emerge as a statistically significant mediator. The indirect effect was −0.069 for the eGFR-anxiety-dopamine pathway (95% CI: −0.154 to −0.015, *p* = 0.107) and for the UACR anxiety-dopamine pathway (95% CI: −0.007 to −0.064, *p* = 0.116). These data indicate that depression serves as a substantial, stable mediator connecting compromised renal functional with diminished dopamine metabolism, whereas the potential mediating role of anxiety lacks statistical reliability.

## 4 Discussion

### 4.1 Principal prospective findings and methodological concordance

This prospective longitudinal investigation demonstrated that somatic wasting phenotypes (sarcopenia and frailty) together with somatopsychic distress (depression and anxiety) were significantly associated with an increased risk of 5-year incident RLS among patients undergoing MHD. Importantly, to minimize fundamental confounding arising from demographic characteristics and dialysis-related infrastructure, an optimal multivariate matching algorithm was retrospectively implemented according to RLS conversion status. This strategy effectively transformed the analysis into a nested case–control comparison within a prospective longitudinal framework, thereby strengthening the temporal sequence between baseline phenotypic exposures and subsequent RLS onset. Following successful baseline homogenization (all SMDs ≤ 0.06), multivariable longitudinal GEE models demonstrated that both sarcopenia and depressive symptom severity independently predicted future RLS development, with their adverse effects progressively amplifying over the 5-year follow-up period. Moreover, higher-order multiplicative interaction analyses identified a pronounced synergistic effect, particularly for the sarcopenia × depression interaction. This compounded risk pattern suggests that concurrent somatic deterioration and psychological distress may converge on shared pathophysiological mechanisms, thereby lowering the threshold for neuro-sensorimotor dysregulation in the setting of advanced uremia.

### 4.2 Survivor bias, temporal precedence, and epidemiological context

A major methodological consideration in hemodialysis cohorts is the inherent vulnerability to selection and survivor biases. In our secondary analyses, restricting the strict longitudinal trajectory modeling to complete follow-up cases risks establishing a healthier-survivor subcohort, given the high background rates of attrition, attrition-driven functional decline, and mortality in ESKD. To provide a rigorous methodological defense against this sample truncation bias, path estimates were replicated across the full unmatched imputed cohort (*N* = 321) utilizing MICE and FIML frameworks, demonstrating structural stability.

Furthermore, although longitudinal data optimize temporal tracking, establishing absolute directional certainty between somatopsychic distress and RLS remains challenging. RLS-induced sleep architecture disruption, nocturnal distress, and motor exhaustion are well-documented drivers of secondary emotional distress and accelerated physical frailty, indicating potential bidirectional feedback loops (34, 35). Nevertheless, because all participants presented with an absolute absence of RLS manifestations at baseline and exposure updates consistently preceded formal annual diagnostic adjudications, our data support the interpretation of these phenotypes as active prospective risk indicators rather than merely concurrent consequences of pre-existing RLS. These results align with broader epidemiological evidence identifying substantial clinical clustering of frailty, malnutrition, and affective disorders within the dialysis population (36–38).

### 4.3 Mechanistic integration, CKM staging, and genetic triangulation

Our study expands upon the traditional cardiovascular-kidney-metabolic syndrome and CKD literature by exploring the “renal dysfunction–psychological vulnerability–RLS” axis. Prior national evidence has increasingly highlighted that advanced renal and cardiometabolic staging contributes profoundly to broader psychological morbidity, insomnia, and adverse neurological outcomes beyond conventional cardiorenal endpoints. Recent large-scale investigations support the conceptualization of depressive and anxiety states as systemic intermediate phenotypes rooted in chronic neuroendocrine dysregulation and microinflammatory uremic toxicity, rather than treating them merely as ordinary statistical covariates (39, 40).

To bridge these clinical observations with downstream neurochemical pathology, it is critical to elucidate the role of dopaminergic dysregulation, a classical hallmark of RLS pathogenesis (41). In human physiology, dopamine undergoes extensive Phase II conjugation, primarily sulfation mediated by sulfotransferases, transforming it into stable metabolites. Among these, plasma dopamine 3-O-sulfate represents the predominant and most metabolically stable sulfated dopaminergic variant in circulation, serving as a highly sensitive and reliable biomarker for capturing global dopaminergic metabolic status. In the context of ESKD, progressive uremic encumbrance and compromised renal clearance profoundly perturb this metabolic pool (6, 42). Although measured peripherally, fluctuations in plasma dopamine 3-O-sulfate reflect a systemic multi-organ metabolic disruption that closely mirrors the neuro-sensorimotor vulnerability and altered dopaminergic tone characteristic of RLS.

Crucially, this investigation is the first to integrate longitudinal clinical modeling with a network mediation MR framework to triangulate these precise neurochemical pathways at the germline level. Our genetic causal inference indicates that lower eGFR causally associates with an increased genetic liability for depression (IVW: *β* = −0.579, *p* = 0.004), which directionally mediates a significant downstream indirect effect (0.401, *p* = 0.004; accounting for 41% of the total effect) on suppressed plasma dopamine 3-O-sulfate levels, whereas anxiety demonstrated low mediating reliability. This implies that genetically predicted renal impairment may suppress systemic dopaminergic metabolic pathways via the specific directional mediation of depressive phenotypes, offering a plausible genetic-level substrate for the somatopsychic-neurological interactions observed in our clinical cohort.

### 4.4 Methodological limitations and diagnostic boundaries

Several inherent limitations must be carefully factored into the interpretation of our data. First, the observational cohort was recruited from a single clinical center, which may restrict the immediate external generalizability of our findings to ethnically or geographically diverse MHD populations. Second, although the separate and decoupled LGCM were rigorously constrained to optimal sample-to-parameter ratios (*N/q* ≥ 10: 1) to guarantee structural equation modeling stability and measurement invariance, the matched sample size (*N* = 192) warrants cautious replication in larger collaborative cohorts. Third, the summary statistics utilized in our MR analyses rely on proxy phenotypes that do not perfectly mirror the actual clinical environment of long-term dialysis. Specifically, population-level GWAS for eGFR and UACR predominantly capture early-to-moderate renal variations rather than the systemic uremic adaptations unique to ESKD (43). Similarly, ALM serves as an indirect genetic proxy for sarcopenia, and peripheral plasma dopamine 3-O-sulfate concentrations cannot be assumed to directly reflect central nervous system dopaminergic function or localized substantia nigra neural pathways.

Finally, we must emphasize that our clinical cohort and genetic MR results represent complementary streams of evidence derived from distinct population scales, exposure intensities, and phenotype definitions. Consequently, these findings should be interpreted as suggesting possible links and prospective associations within a plausible mechanistic hypothesis, rather than as a fully established, direct causal chain. Definitive neurobiological validation is still lacking, as our protocol did not incorporate direct central nervous system dopamine measurements, cerebral iron markers (e.g., magnetic resonance phase imaging), polysomnographic sleep tracking, or cerebrospinal fluid biomarker assays.

### 4.5 Clinical implications and future directions

In conclusion, while keeping these diagnostic boundaries in mind, our data suggest that the management of MHD-associated RLS should transcend isolated cardiorenal or iron-supplementation parameters. Clinical intervention strategies must prioritize early multi-system screening, actively targeting the maintenance of musculoskeletal performance and the proactive management of psychological well-being. Evolving therapeutic models should evaluate whether timely, multi-disciplinary interventions targeting depression and sarcopenia can attenuate the long-term cumulative trajectory leading to secondary neuro-sensorimotor dysregulation, thereby improving the long-term survival and holistic quality of life for patients undergoing maintenance dialysis.

## 5 Conclusion

In conclusion, this study demonstrates a prospective, time-dynamic clustering of somatic wasting and somatopsychic vulnerability that independently and synergistically predicts 5-year incident RLS in MHD patients. By integrating longitudinal cohort data with a network mediation MR framework, our findings suggest a plausible, genetically reinforced “somatopsychic-neurologica” cascade. Within this paradigm, chronic phenotypic deterioration and genetically predicted depression—rather than anxiety—serve as intermediate pathways linking renal impairment to suppressed systemic dopaminergic metabolism (proxied by plasma dopamine 3-O-sulfate). While direct central neurobiological validation is required, these insights suggest that MHD-associated RLS management should transcend conventional cardiorenal and iron parameters, prioritizing early multi-systemic screening and multi-disciplinary interventions targeting musculoskeletal performance and psychological well-being.

## Data Availability

The raw data supporting the conclusions of this article will be made available by the authors, without undue reservation.

## Glossary

RLS: Restless Legs Syndrome
CKD: Chronic Kidney Disease
MHD: Maintenance Hemodialysis
GEE: Generalized Estimating Equations
LGCM: Latent Growth Curve Mediation
MR: Mendelian Randomization
GWAS: Genome-Wide Association Study
eGFR: estimated Glomerular Filtration Rate
UACR: Urinary Albumin-To-Creatinine Ratio
BMI: Body Mass Index
AVF: Arteriovenous Fistula
CCI: Charlson Comorbidity Index
ESA: erythropoiesis-Stimulating Agents
CRP: C-Reactive Protein
iPTH: Intact Parathyroid Hormone
SMI: Skeletal Muscle Mass Index
HAMD-17: 17-item Hamilton Depression Rating Scale
GAD-7: 7-item Generalized Anxiety Disorder Scale
FIML: Full Information Maximum Likelihood
MICE: Multiple Imputation by Chained Equations
AWGS 2019: Asian Working Group for Sarcopenia: 2019 consensus update on sarcopenia diagnosis and treatment
SMD: Standardized Mean Difference
C-index: Concordance Index
CFA: Confirmatory Factor Analysis
CIs: Confidence Intervals
ALM: Appendicular Lean Mass
FI: Frailty Index
IVW: Inverse Variance Weighted
