# Supplementary material for: Risk factors and mediating mechanisms of restless legs syndrome in patients undergoing maintenance hemodialysis: a longitudinal cohort study combined with Mendelian randomization analysis
Source: Front Neurol. 2026 Jul 2;17:1819747. doi: 10.3389/fneur.2026.1819747 (PMC13372772; doi:10.3389/fneur.2026.1819747)
Supplement: Supplementary file 1 [file Data_Sheet_1.docx]

**Risk Factors and Mediating Mechanisms of Restless Legs Syndrome in Patients Undergoing Maintenance Hemodialysis: A Longitudinal Cohort Study Combined with Mendelian Randomization Analysis**

**Supplement**

**Table S1.** Overview of GWAS datasets used for two-step mediation Mendelian randomization analyses in this study

| **GWAS ID** | **Trait** | **Years** | **population** | **sample_**  **size** | **consortium** | **nsnp** |
| --- | --- | --- | --- | --- | --- | --- |
| GCST90265057 | Dopamine 3-o-sulfate levels | 2023 | European | 4, 948 | / | 7, 724, 597 |
| GCST90000025 | ALM | 2020 | European | 450,243 | UKB | 8,391,926 |
| ukb-b-12064 | Depression | 2018 | European | 462, 933 | MRC-IEU | 9, 851, 867 |
| GCST90020053 | FI | 2021 | European | 175,226 | UKB，TwinGene | 7,663,022 |
| ukb-a-82 | Anxiety | 2017 | European | 337, 159 | Neale Lab | 10, 894, 596 |
| ieu-a-1284 | eGFR | 2018 | East Asian | 143, 658 | Biobank Japan Project | 596, 1601 |
| ieu-a-1107 | Urinary albumin-to-creatinine ratio | 2015 | European | 54, 450 | CKDGen | 219, 0189 |

**Note:** The table lists the genome-wide association study datasets used in this study for two-step mediation Mendelian randomization analyses, including the GWAS identifier (GWAS ID), trait name (Trait), publication year (Years), study population (Population), sample size, contributing consortium or data source (Consortium), and the number of SNPs (nSNP) selected for analysis. **Abbreviations:** ALM, Appendicular Lean Mass; FI, Fat Index; eGFR, estimated Glomerular Filtration Rate; UACR, Urinary Albumin-to-Creatinine Ratio.

**Table S2.** Negative Control Outcome Analysis (GEE Model)

| **Exposures** | **Negative Control Outcome** | **OR (95% CI)** | **P value** |
| --- | --- | --- | --- |
| Sarcopenia | Negative Control 1 | 1.05 (0.89–1.24) | 0.58 |
| Frailty | Negative Control 1 | 1.02 (0.88–1.18) | 0.76 |
| Depression (HAMD‑17 > 7) | Negative Control 1 | 1.08 (0.92–1.27) | 0.35 |
| Anxiety (GAD‑7 > 5) | Negative Control 1 | 1.03 (0.88–1.20) | 0.7 |

**Notes:** Data are presented as multivariable-adjusted ORs with corresponding 95% CIs, estimated using longitudinal GEE models.

1. **Statistical Specification:** To rigorously evaluate the potential influence of unmeasured confounding and systematic bias, a negative control outcome analysis was conducted. The GEE models were constructed using the same specifications as the primary multivariable analysis and were fully adjusted for prespecified baseline covariates, including age, sex, BMI, dialysis vintage, vascular access type, dialysis adequacy (Kt/V), and Charlson Comorbidity Index. Negative Control 1 represented a theoretically unrelated clinical endpoint with no biologically plausible causal association with the investigated exposures.
2. **Methodological Validity:** The absence of statistically significant prospective associations (all *P* > 0.05) between the core phenotypic exposures and the negative control outcome suggests that the primary findings are unlikely to be substantially influenced by residual confounding or structural model artifacts, thereby supporting the methodological robustness of the main analyses.

**Abbreviations:** BMI, body mass index; CI, confidence interval; GEE, generalized estimating equations; GAD-7, 7-item Generalized Anxiety Disorder scale; HAMD-17, 17-item Hamilton Depression Rating Scale; OR, odds ratio.

**Table S3.** Sensitivity Analysis of Latent Growth Curve Mediation Models Based on the Full Unmatched Cohort with Multiple Imputation (*‬N* = 321)

| **Mediator (Latent Construct)** | **Latent Intercept (I)**  **β(SE)** | **Latent Slope (S)**  **β(SE)** | **Direct Effect**  **β (SE)** | **Indirect Effect**  **β (SE)** | **Total Effect**  **β (SE)** | **P value** | **Multi-Model Fit Indices** |
| --- | --- | --- | --- | --- | --- | --- | --- |
| Depression | 0.19 (0.04) | 0.16 (0.04) | 0.14 (0.03) | 0.10 (0.02) | 0.24 (0.03) | <0.001 | CFI=0.958, RMSEA=0.049, SRMR=0.039 |
| Anxiety | 0.15 (0.03) | 0.13 (0.03) | 0.11 (0.03) | 0.09 (0.02) | 0.20 (0.03) | <0.001 | CFI=0.961, RMSEA=0.044, SRMR=0.036 |
| Frailty | 0.17 (0.04) | 0.15 (0.04) | 0.12 (0.03) | 0.10 (0.02) | 0.22 (0.03) | <0.001 | CFI=0.952, RMSEA=0.052, SRMR=0.041 |
| Sarcopenia | 0.20 (0.05) | 0.16 (0.04) | 0.13 (0.03) | 0.11 (0.03) | 0.24 (0.03) | <0.001 | CFI=0.955, RMSEA=0.048, SRMR=0.037 |

**Notes:** Data are presented as standardized path coefficients (*β*‬) with their corresponding SE.

1. ****Sensitivity Framework:**** To test the robustness of the primary findings against potential selection bias from clinical matching, this sensitivity analysis was conducted using the full unmatched cohort (*N* = 321‬). Missing data were handled using MICE based on 20 replicated datasets.
2. ****Model Configurations:**** Four separate latent growth curve mediation models were constructed following identical architectural logic to the primary analysis, with cumulative dialysis duration as the independent variable (*X*‬), and latent intercepts (*I*‬) and latent slopes (*S*‬) of each phenotype serving as parallel mediators (*M*‬) toward incident RLS (*Y*‬).
3. ****Statistical Validity:**** Each independent framework demonstrated acceptable global fit criteria (all CFI ≥ 0.952, RMSEA ≤ 0.052, SRMR ≤ 0.041‬‬). The consistency of both direct and indirect effect distributions across the unmatched cohort indicates that the primary matched-sample findings are less likely to be biased by sample truncation.

**Abbreviations:** *β*‬, standardized path coefficient; CFI, comparative fit index; RLS, restless legs syndrome; RMSEA, root mean square error of approximation; SE, standard error; SRMR, standardized root mean square residual.

**Table S4.** Heterogeneity and horizontal pleiotropy statistics for all Mendelian randomization analyses

| **Exposure** | **0utcome** | **Heterogeneity test** | | | | **Horizontal pleiotropy test** | |
| --- | --- | --- | --- | --- | --- | --- | --- |
|  |  | **IVW** | | **MR Egger** | | **MR Egger** | |
|  |  | **Cochran’s Q** | **P** | **Cochran’s Q** | **P** | **Intercept** | **P** |
| eGFR | Depression | 82.514 | 0.096 | 82.508 | 0.0824 | 0.0002 | 0.746 |
|  | Anxiety | 57.26 | 0.863 | 57.701 | 0.872 | 0.0001 | 0.506 |
|  | ALM | 53.41 | 0.633 | 58.612 | 0.702 | 0.0022 | 0.409 |
|  | FI | 68.875 | 0.448 | 69.142 | 0.473 | -0.005 | 0.609 |
|  | Dopamine 3-o-sulfate | 50.512 | 0.424 | 51.421 | 0.456 | 0.0014 | 0.452 |
| UACR | Depression | 65.92 | 0.171 | 66.201 | 0.189 | 0 | 0.627 |
|  | Anxiety | 63.103 | 0.269 | 63.092 | 0.24 | 0.0036 | 0.923 |
|  | ALM | 77.292 | 0.254 | 77.296 | 0.312 | 0.0061 | 0.958 |
|  | FI | 76.908 | 0.333 | 78.41 | 0.031 | -0.0009 | 0.3 |
|  | Dopamine 3-o-sulfate | 61.485 | 0.286 | 62.052 | 0.301 | -0.0061 | 0.476 |
| Depression | Dopamine 3-o-sulfate | 46.278 | 0.168 | 46.3 | 0.196 | 0.019 | 0.897 |
| Anxiety |  | 32.891 | 0.842 | 34.767 | 0.81 | 0.0163 | 0.178 |
| ALM |  | 63.081 | 0.43 | 62.451 | 0.506 | -0.0046 | 0.772 |
| FI |  | 94.327 | 0.101 | 95.122 | 0.104 | 0.092 | 0.42 |

**Notes:** Cochran’s Q‬ statistics and corresponding *P*‬ values estimated via IVW and MR-Egger regression models are presented to rigorously assess potential heterogeneity among the selected instrumental variables.

1. ****Pleiotropy and Validity Diagnostics:**** Directional horizontal pleiotropy was evaluated using the MR-Egger intercept test, with intercept estimates and *P*‬ values reported. Statistically significant heterogeneity or horizontal pleiotropy was defined under a threshold of *P <* 0.05‬.
2. ****Framework Purpose:**** These diagnostic evaluations were conducted as an integral part of the comprehensive sensitivity framework to validate the exclusion restriction assumption of the instrumental variables across all exposure–outcome pathways involved in the two-step mediation Mendelian randomization analyses

**Abbreviations:** eGFR, estimated glomerular filtration rate; UACR, urinary albumin-to-creatinine ratio; ALM, appendicular lean mass; FI, fat index; IVW, inverse variance weighted.

**Figure**

|  |  |  |  |
| --- | --- | --- | --- |
| A | B | C | D |
|  |  |  |  |
| E | F | G | H |

**Figure S1.** Two-step mediation Mendelian randomization analyses linking kidney function, body composition traits, and dopamine metabolite levels

**Note:** Scatter plots illustrating genetic causal estimates derived from two-step mediation MR analyses evaluating the relationships among kidney function indicators, body composition traits, and dopamine metabolite levels. Each point represents the SNP-specific association with the exposure (x-axis) and the corresponding association with the outcome (y-axis). Solid lines denote causal estimates obtained using complementary MR approaches, including IVW, MR-Egger regression, weighted median, simple mode, and weighted mode methods, enhancing robustness against horizontal pleiotropy and heterogeneity.

**Step 1 (exposure → mediator):**

**(A)** Genetically predicted eGFR and ALM;

**(B)** Genetically predicted eGFR and FI;

**(C)** Genetically predicted eGFR and Dopamine 3-O-sulfate levels;

**(D)** Genetically predicted UACR and ALM;

**(E)** Genetically predicted UACR and FI;

**(F)** Genetically predicted UACR and Dopamine 3-O-sulfate levels.

**Step 2 (mediator → outcome):**

**(G)** Genetically predicted ALM and Dopamine 3-O-sulfate levels;

**(H)** Genetically predicted FI and Dopamine 3-O-sulfate levels.

Collectively, these analyses delineate a putative genetic pathway whereby renal dysfunction may influence dopaminergic metabolism directly and indirectly through alterations in body composition traits.

**Abbreviations:** eGFR, estimated glomerular filtration rate; UACR, urinary albumin-to-creatinine ratio; ALM, appendicular lean mass; FI, fat index.

|  |  |  |  |
| --- | --- | --- | --- |
| A | B | C | D |
|  |  |  |  |
| E | F | G | H |

**Figure S2**. Leave-one-out sensitivity analyses for two-step mediation Mendelian randomization models linking kidney function, body composition traits, and dopamine metabolite levels

**Note:** Forest plots presenting leave-one-out sensitivity analyses for the two-step mediation MR models. Each point represents the causal estimate obtained after sequential removal of a single SNP from the instrumental variable set using the IVW method. Horizontal lines indicate the corresponding 95% confidence intervals. The vertical line denotes the overall causal estimate derived from the full SNP set.

These analyses assess whether the observed associations are disproportionately driven by any single instrumental variant and evaluate the robustness and stability of the estimated mediation pathway.

**Step 1 (exposure → mediator/outcome):**

**(A)** eGFR on ALM;

**(B)** eGFR on FI;

**(C)** eGFR on Dopamine 3-O-sulfate levels;

**(D)** UACR on ALM;

**(E)** UACR on FI;

**(F)** UACR on Dopamine 3-O-sulfate levels.

**Step 2 (mediator → outcome):**

**(G)** ALM on Dopamine 3-O-sulfate levels;

**(H)** FI on Dopamine 3-O-sulfate levels.

The consistency of effect estimates across iterations supports the stability of the instrumental variables and strengthens confidence in the inferred genetic mediation pathway.

**Abbreviations:** eGFR, estimated glomerular filtration rate; UACR, urinary albumin-to-creatinine ratio; ALM, appendicular lean mass; FI, fat index.

|  |  |  |  |
| --- | --- | --- | --- |
| A | B | C | D |
|  |  |  |  |
| E | F | G | H |

**Figure S3.** Funnel plots assessing horizontal pleiotropy in two-step mediation Mendelian randomization analyses linking kidney function, body composition traits, and dopamine metabolite levels

**Note:** Funnel plots displaying SNP-specific causal estimates against their precision for the two-step mediation MR models. Each dot represents an individual SNP. The horizontal axis shows the causal effect estimate, and the vertical axis represents the precision (inverse of the standard error). The vertical lines correspond to the pooled causal estimates derived from IVW and MR-Egger methods.

Symmetry of the funnel plots around the overall causal estimate suggests the absence of substantial directional horizontal pleiotropy or small-study effects. These visual assessments complement MR-Egger intercept tests and other sensitivity analyses to evaluate the validity of instrumental variables.

**Step 1 (exposure → mediator/outcome):**

**(A)** eGFR on ALM;

**(B)** eGFR on FI;

**(C)** eGFR on Dopamine 3-O-sulfate levels;

**(D)** UACR on ALM;

**(E)** UACR on FI;

**(F)** UACR on Dopamine 3-O-sulfate levels.

**Step 2 (mediator → outcome):**

**(G)** ALM on Dopamine 3-O-sulfate levels;

**(H)** FI on Dopamine 3-O-sulfate levels.

Overall symmetry supports the robustness of the instrumental variables and reduces the likelihood that the observed associations are driven by directional pleiotropy.

**Abbreviations:** eGFR, estimated glomerular filtration rate; UACR, urinary albumin-to-creatinine ratio; ALM, appendicular lean mass; FI, fat index.
